# Force transduction by cadherin adhesions in morphogenesis

**DOI:** 10.12688/f1000research.18779.1

**Published:** 2019-07-10

**Authors:** Willem-Jan Pannekoek, Johan de Rooij, Martijn Gloerich

**Affiliations:** 1 Molecular Cancer Research, Center for Molecular Medicine, University Medical Center Utrecht, Utrecht, The Netherlands

**Keywords:** E-cadherin, adherens junction, mechanical force, mechanotransduction, collective migration, spindle orientation, intercalation

## Abstract

Mechanical forces drive the remodeling of tissues during morphogenesis. This relies on the transmission of forces between cells by cadherin-based adherens junctions, which couple the force-generating actomyosin cytoskeletons of neighboring cells. Moreover, components of cadherin adhesions adopt force-dependent conformations that induce changes in the composition of adherens junctions, enabling transduction of mechanical forces into an intracellular response. Cadherin mechanotransduction can mediate reinforcement of cell–cell adhesions to withstand forces but also induce biochemical signaling to regulate cell behavior or direct remodeling of cell–cell adhesions to enable cell rearrangements. By transmission and transduction of mechanical forces, cadherin adhesions coordinate cellular behaviors underlying morphogenetic processes of collective cell migration, cell division, and cell intercalation. Here, we review recent advances in our understanding of this central role of cadherin adhesions in force-dependent regulation of morphogenesis.

## Introduction

Morphogenesis comprises the collection of processes that shape tissues and organisms, which relies on the coordinated regulation of cell behavior. Though most apparent throughout embryonic development, morphogenetic processes continue to play a role in adult tissues (for instance, during tissue regeneration upon wounding as well as pathological conditions such as cancer invasion). Morphogenesis inherently is a mechanical process, as the coordinated generation of forces by cells is required to establish tissue shape ^1, 2^. In addition, forces exerted on cells by each other and their surroundings serve as instructive signals to which cells adapt their behavior. This depends on the ability of cells to sense and convert mechanical forces into a biochemical intracellular response (termed mechanotransduction), which is mediated by force-induced changes in the conformation of proteins and the composition of molecular complexes ^3, 4^. Mechanical forces typically originate from contraction of the actomyosin cytoskeleton, which is anchored to sites of adhesion and can thereby be transmitted to neighboring cells and the extracellular environment. As such, focal adhesions (at the cell–matrix interface) and adherens junctions (AJs) (at the cell–cell interface) represent key force transmission and transduction complexes of the cell. In recent years, many additional components of the cell cortex, including growth factor receptors, ion channels, caveolae, and the glycocalyx, have been identified to be responsive to forces ^5^. In this review, we will focus on AJs, as they have emerged as central mechano-responsive components in the regulation of morphogenesis by intercellular forces.

## Mechanotransduction by adherens junctions

**Figure 1.**
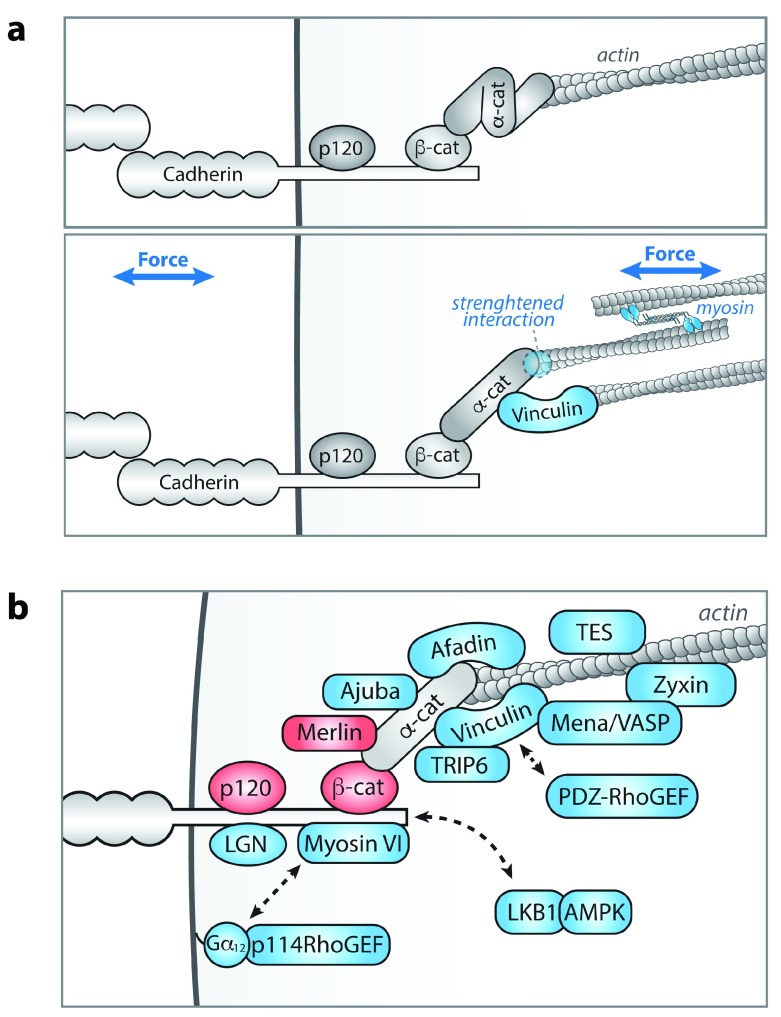
Mechanotransduction by the cadherin complex. ( **a**) Tensile forces on the cadherin complex, exerted either by neighboring cells through its extracellular domain or by myosin-generation contraction of the actin cytoskeleton associated with its cytosolic tail, cause unfolding of α-catenin, enabling its interaction with Vinculin. The interaction between α-catenin and F-actin is regulated by force as well, as β-catenin–bound α-catenin forms a catch-bond interaction with F-actin that shifts from a weakly bound to strongly bound state with increasing tension. Mechanical force can also influence transdimer interactions formed by the extracellular domain of E-cadherin, as X-dimer configurations of the transdimer form a catch-bond interaction whose lifetime increases with force (not shown) ^37^. ( **b**) Numerous proteins that are recruited to cadherin adhesions in a tension-dependent manner have been identified and show either increased (blue) or decreased (red) association with the cadherin complex upon increased tensile force. Dashed lines indicate tension-dependent adherens junction (AJ) components of which the molecular mechanism underlying association with the cadherin complex has not yet been resolved. α-cat, α-catenin; β-cat, β-catenin; p120, p120-catenin.

The AJ is composed of transmembrane cadherin proteins (E-cadherin being the predominant cadherin in most epithelial tissues) that form homotypic dimers between neighboring cells *.* The cytosolic tail of cadherins compiles a large protein complex, which dynamically connects to the actomyosin cytoskeleton ^6^. The core cadherin complex consists of p120-catenin, which controls cadherin membrane localization, and β-catenin and α-catenin, which provide a link with filamentous actin ( Figure 1a). Importantly, β-catenin–bound α-catenin forms a catch-bond interaction with F-actin that is strengthened when the complex experiences tensile force ( Figure 1a) ^7^, which is explained by the force-dependent exposure of a cryptic actin-binding site within α-catenin ^8^. In addition to enhanced actin binding of α-catenin, other force-induced changes occur in the cadherin complex that regulate the organization of junctional actin, thereby reinforcing cadherin junctions. Most well studied is the exposure of a binding site within α-catenin for Vinculin ( Figure 1a), which provides additional linkage of the cadherin complex with actin ^9– 12^ and recruits several actin-modulating proteins ^13, 14^. Intriguingly, Vinculin was recently shown to form a force-stabilized linkage to F-actin that is directionally asymmetric ^15^, suggesting that Vinculin may further contribute to junctional actin remodeling by organizing the polarity of actin filaments. In addition to Vinculin, numerous other actin assembly and remodeling proteins are recruited when the cadherin complex is under tension upon application of forces from neighboring cells or the actin cytoskeleton ( Figure 1b). These bind either directly to α-catenin (Afadin ^16^) or indirectly through other junctional components or the actin cytoskeleton (RhoGEF114 ^17^, VASP ^18^, TES ^18^, and Zyxin ^18^). This large number of identified actin-associated proteins recruited to tensed cadherin junctions confers several layers of mechanical control of the AJ–actin link, potentially explaining the mild phenotype of selectively losing Vinculin in various tissues ^19– 21^.

By mechanically coupling the actomyosin network of neighboring cells, cadherin adhesions are essential for the transmission of forces between individual cells. As a result, cell within tissues develop collective contractility that allows them to coordinate morphogenetic cell rearrangements in tissues ^2, 22, 23^. Moreover, the different mechanisms to transduce force into regulation of the organization of the actin cytoskeleton and its association with the cadherin complex strengthen cell–cell adhesions, enabling them to maintain integrity of tissues upon fluctuations in intercellular stresses. However, the cellular responses to junctional force reach much further, impacting, for instance, the cell cycle and cell division ^24, 25^, cell migration ^26, 27^, and cell metabolism ^28^. This is mediated by force-regulated association of the cadherin complex with transcriptional activators (for example, β-catenin and YAP ^24, 29^), kinases (for example, LKB1/AMPK ^28^), and interactors of microtubules and intermediate filaments ( Figure 1b) ^25, 26^. E-cadherin thus should be envisioned not as a static mediator of cell–cell adhesion but rather as a dynamic sensor of tensile forces that instructs cellular behavior. In this way, E-cadherin fulfills a central role in transducing cellular forces to coordinate morphogenesis, as it instructs several processes that direct tissue shape, including collective migration, tissue growth, and cell–cell intercalation. Below, we will discuss recent advances in our understanding of the role of E-cadherin in the force-dependent regulation of these morphogenetic processes.

## Cadherin mechanotransduction in collective migration

Tissue rearrangement during morphogenesis can be achieved by collective migration of groups or sheets of cells, which move while retaining adhesion between individual cells. These adhesions enable cell-to-cell communication to coordinate the direction of motion. As a result, cell clusters migrate more efficiently and persistently than isolated cells ^30^. Moreover, migrating as a collective enables epithelial clusters to follow specific guidance cues while single cells or monolayers lacking AJs fail to do so ^27, 31– 33^. It has been well established that cadherin-based adhesions enable collective migration by coupling cells to one another and by transmitting forces between cells to convey directional information (reviewed in 30, 34, 35). In migrating sheets, cells at the edge generate lamellipodia that protrude into the surrounding environment. These lamellipodia adhere to the surface and generate traction forces required for forward pulling of the cells ^30, 35^. Importantly, the pulling forces are exerted not only by these cells but by all cells in the cell sheet, which is coordinated via intercellular force transmission ^36^. Cadherin junctions are the main force transducer between cells responsible for this, as the correlation between the orientations of migration and intercellular forces is lost by blocking E-cadherin function or depleting individual AJ components ^38, 39^. The migration direction of individual cells in a monolayer follows the local axis of maximum intercellular stresses ^40^. More recently, it was shown that tension on AJs is anisotropic and highest at cell–cell contacts perpendicular to the migration direction ^16^. This implies that forces acting locally at cell–cell contacts may help to establish the direction of motion. Indeed, experiments with α-catenin mutants in which Vinculin binding is perturbed, showed that transduction of forces by AJs is essential for coordinated motion in epithelial monolayers ^16, 41^. These results are corroborated *in vivo*, as shown by the convergence-extension defects observed in zebrafish expressing Vinculin binding–deficient α-catenin ^42^.

**Figure 2.**
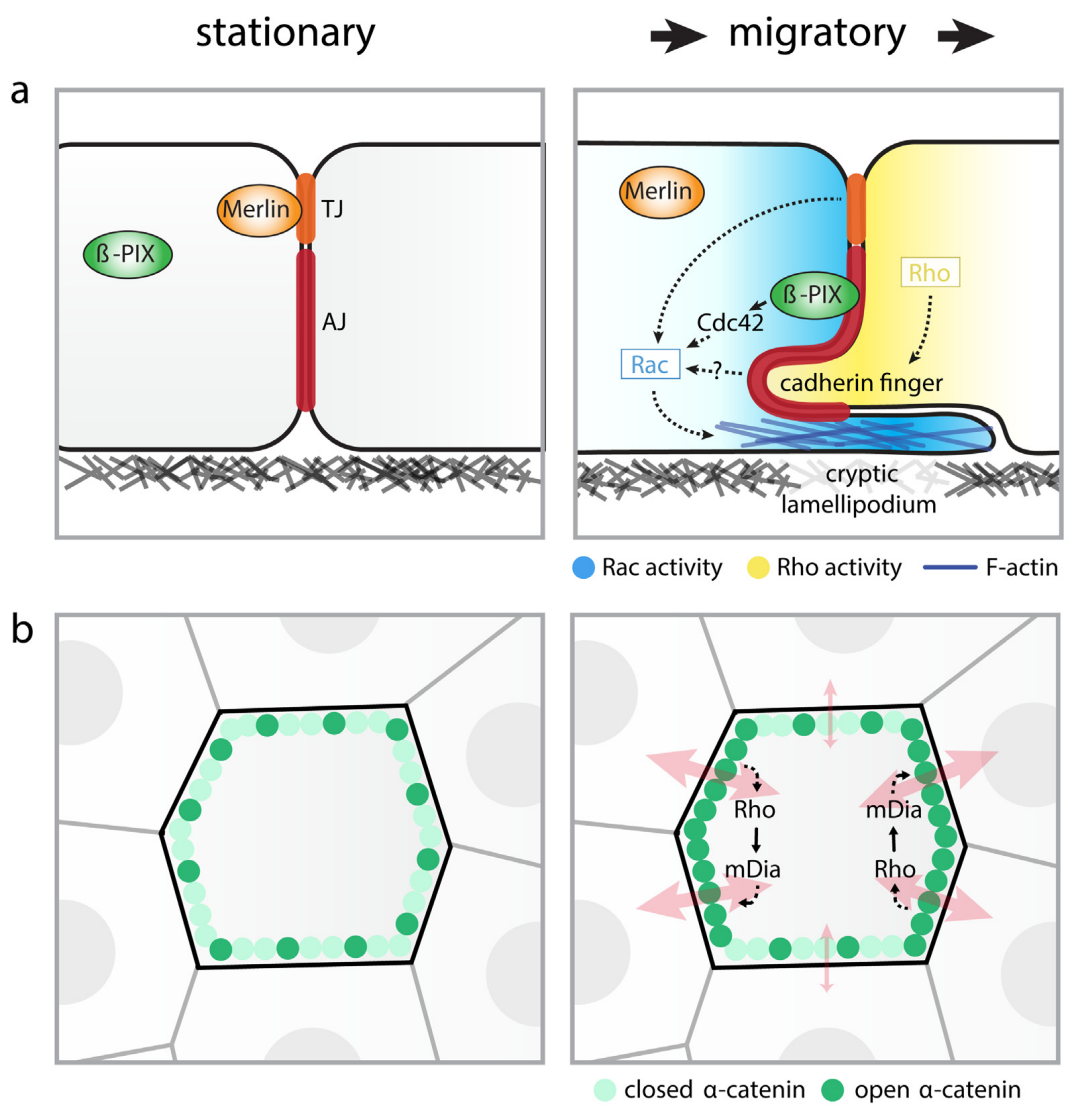
Mechanotransduction at cell – cell junctions during collective cell migration. ( **a**) Mechanotransduction at cell–cell junctions enables cells to coordinate cell polarization with their followers via several mechanisms that impinge on local activation of Rac, which establishes the formation of (cryptic) lamellipodia: (1) Tension on cell–cell junctions causes loss of Merlin from tight junctions (TJ), relieving its inhibition of Rac activation; (2) β-PIX is recruited by P-cadherin to adherens junctions (AJs) in migrating cells, presumably in a force-dependent manner, which results in activation of Cdc42 and downstream Rac1 to promote cell polarization; (3) Rho-mediated contractility at the rear of migrating cells induces the formation of cadherin fingers that extend into the trailing cells, which direct the formation of cryptic lamellipodia, presumably by contributing to the control of Rac activity. ( **b**) During collective migration, force-dependent conformational opening of α-catenin occurs in an anisotropic manner and is highest at cell–cell junctions perpendicular to the migratory axis. This involves a feedback mechanism through α-catenin–mediated activation of Rho and its effector mDia that induces AJ remodeling and junction strengthening. Anisotropy in the force-dependent remodeling of AJs coordinates the direction of migration between cells, possibly by establishing preferential transmission of forces between cells in the direction of migration (red arrows).

To establish directionality of collective migration, AJs not only transmit forces between cells but also transduce these forces into a cellular response to guide migration. Essentially, cell migration relies on the acquisition of a polarized cell state with Rac-induced (cryptic) lamellipodia protruding forward and Rho-mediated adhesion disassembly at the rear ^43^. Accumulating evidence shows that force transduction at cell–cell junctions enables cells to coordinate this polarization and concomitant migration direction to their followers ( Figure 2a) ^16, 27, 44^. This is mediated, at least in part, by the force-induced release of Merlin from cell contacts, which induces polarized Rac activity and formation of cryptic lamellipodia in migrating epithelial layers ^44^. While force-induced release of Merlin from tight junctions is implicated in this ^44^, the association of Merlin with the cadherin complex was recently shown to be inhibited by tensile forces as well ^45^. In migrating border cells in the *Drosophila* egg chamber, E-cadherin also transduces mechanical forces into Rac activation, resulting in protrusive activity at cell–cell contacts that are under elevated tension ^27^. Ectopic expression of P-cadherin in migrating myoblasts further revealed that the Cdc42-GEF β-PIX is recruited to AJs in motile cells, presumably as a result of increased junctional tension ^46^. This establishes the activation of Cdc42 and downstream Rac, thereby coordinating cell polarity ^46^. The selective function of P-cadherin, and not other cadherins, in β-PIX regulation highlights that different cadherin proteins may have distinct functions in transducing mechanical forces to establish collective cell migration. In line with this, E- and P-cadherin were shown to have distinct contributions to the development of intercellular stresses in migrating epithelia ^39^.

In addition to coordinating front–rear polarity, activation of Rho-family GTPases provides a positive feedback mechanism by reinforcing tension on E-cadherin junctions ^16, 27^. Conformational opening of α-catenin induces local activation of Rho and downstream mDia-mediated actin remodeling, which strengthens anisotropy in junctional tension in migrating epithelia ( Figure 2b) ^16^. Polarized Rho activity can also couple directional information between cells through the formation of specialized adhesion structures called cadherin fingers ( Figure 2a). At the rear of migrating endothelial cells, local RhoA-induced contractility triggers the formation of cadherin fingers, which become engulfed in follower cells and direct the formation of cryptic lamellipodia. The highly curved membrane in the cadherin finger is potentially instructive for Rac activation in follower cells ^47^, as many small GTPase regulators contain membrane curvature-recognizing domains ^48^. As such, an important signaling cue can be induced by tension-mediated membrane curvature at the cell–cell junction interface ^49^.

Although the mechanisms of force transmission and transduction by AJs discussed above apply, in principle, to all cells in a migrating group, additional mechanisms may be at play at the migratory front. Migration is typically directed by a specified leader cell that is morphologically and functionally distinct from the rest of the cell population and that initiates and organizes the migration of follower cells ^50^. Importantly, polarization and directed migration of the leader cell are regulated by transduction of forces at the cell–cell contact with its followers (reviewed in 30, 51). Moreover, it was recently shown that tensile forces exerted by follower cells on prospective leaders induce their leader cell phenotype ^52^. Another active function of the followers during collective migration was recently revealed for the very rear cells of the migrating cluster of neural crest cells ^53^. Reminiscent of the purse-string mechanism of wound closure, these cells contract a supracellular actin cable that causes the cell cluster to be propelled forward ^53^. Since both of these recently identified functions of follower and rear cells critically depend on force coordination between cells, it will be interesting to establish whether and how cadherin junctions contribute to this.

## Mechanical control of tissue growth

The development of tissues into their correct size and proportions requires tight control of tissue growth and thus the rate and pattern of cell proliferation. Both of these are tightly regulated by, among other signals, mechanical forces that cells exert on each other. Seminal experiments in cultured monolayers revealed that patterns of local proliferation strongly correlate with the level of cellular traction forces ^54^. This was shown to be dependent on force transmission by cadherin-based adhesions ^54^, and specific analyses of intercellular forces demonstrated that the tendency of cells to proliferate correlates with the level of junctional tension ^55, 56^. Importantly, as tissues increase in cell density, junctional tension becomes reduced ^57^ because of a decrease in cell motility and cortical actomyosin contractility ^57, 58^. As such, sensing of intercellular tension provides a mechanism for cells to regulate density-dependent proliferation. At high cell density, proliferation ceases and this can be relieved by application of external stretch, as shown in both cultured cells ^24, 59– 61^ and the *Drosophila* wing disc ^62^. In these experiments, mechanical stretch was shown to control proliferation by inducing cell cycle entry of quiescent cells ^24, 59, 61^ as well as promoting progression through the subsequent cell cycle phases ^24, 60, 63^.

Thus far, the contribution of tension-regulated proliferation to morphogenesis has been studied mainly in the developing *Drosophila* wing disc, in which transduction of junctional tension is directly linked to regulation of organ size ^57, 64^. Moreover, mechano-sensitive control of proliferation in the wing disc explains how developing tissues can maintain homogenous patterns of proliferation despite the presence of gradients of growth factors. In the center of the wing disc, high levels of growth factor–induced proliferation and concomitant increase in cell density decrease junctional tension ^57, 65^. This feeds back to reduce proliferation in this region and thereby balances local proliferation rates throughout the tissue to maintain tissue organization ^66^. More recently, transduction of mechanical forces was also shown to regulate tissue size after development and, for instance, may adapt the size and composition of the *Drosophila* midgut to the feeding state of the animal ^67, 68^.

**Figure 3.**
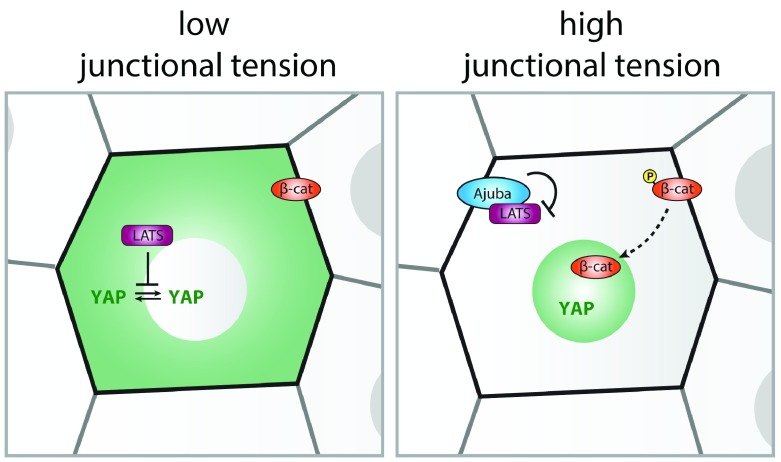
Regulation of YAP and β-catenin mediated transcription by junctional tension. Phosphorylation by LATS establishes the nuclear exclusion of the transcriptional activator YAP under conditions of low junctional tension. The conformational opening of α-catenin upon elevated tension on E-cadherin adhesions (for instance, at decreased cell density) enables junctional recruitment of Ajuba-family proteins. Ajuba sequesters LATS at cell–cell contacts and keeps it inactive at this site, allowing nuclear entry of YAP. Also, the related Zyxin-family protein TRIP6 localizes to AJs, through an interaction with Vinculin, and binds and inhibits LATS (not shown). Note that additional LATS-independent mechanisms have been proposed for the intercellular tension-dependent regulation of YAP (see text). The nuclear localization of the transcriptional coactivator β-catenin is also regulated by the level of tension on cadherin adhesions. Upon increased junctional tension, a subset of the junctional β-catenin pool is phosphorylated (on Y654 in mammals), resulting in its dissociation from E-cadherin. This promotes translocation of β-catenin into the nucleus to drive TCF-dependent gene transcription. Note that loss of tension on E-cadherin has also been shown to promote junctional release and transcriptional activity of β-catenin, independent of phosphorylation of Y654 ^77^.

Although additional mechano-sensing complexes may be involved, E-cadherin adhesions play a key role in transducing intercellular forces to the cell cycle. It has long been established that the presence of E-cadherin adhesions can inhibit proliferation by triggering cell cycle exit, a process termed contact inhibition of proliferation ^69^. This involves inhibition of growth signals by E-cadherin adhesions, most notably through regulation of the Hippo signaling pathway ^70^. The Hippo pathway consists of a cascade of kinases that ultimately establishes the nuclear exclusion of the transcriptional activator YAP (and its homologue TAZ) through phosphorylation by LATS kinases ^71^. As recently demonstrated in the *Drosophila* wing disc and cultured epithelia, increased tension on E-cadherin junctions (for instance, by decreasing cell density or externally applied mechanical stretch) relieves this negative regulation, thereby promoting nuclear localization of YAP and cell cycle entry ^24, 57, 72, 73^. This is explained by force-induced opening of α-catenin that enables its interaction with proteins of the Ajuba/Zyxin family, which sequester LATS in an inactive state at AJs ( Figure 3) ^57, 64, 72, 73^. Several additional mechanisms may further contribute to the regulation of YAP upon fluctuations in tension on cadherin junctions ^71^. These may in part regulate YAP independently of the Hippo pathway and impinge on sequestration of YAP itself at cadherin junctions ^74, 75^, modulate nuclear import by influencing nuclear pore dynamics ^34^, attenuate nuclear export ^45, 76^, or influence YAP through modulation of the cortical cytoskeleton ^59, 78^. In addition to YAP signaling, force transduction by E-cadherin junctions can influence tissue growth through regulation of other proliferation pathways such as EGFR-mediated signaling ^79, 80^ and β-catenin/TCF-mediated transcription. The molecular mechanism of the latter is best understood as mechanical tension on the cadherin complex induces phosphorylation of β-catenin on Y654 ^29, 81^. This site may become increasingly exposed upon elevated junctional tension, and phosphorylation results in junctional dissociation of β-catenin to promote its translocation to the nucleus and TCF-mediated transcription ^82^. Force-induced TCF activation has been linked to G _1_/S progression in mechanically stretched epithelial cultures ^24^. Moreover, activation of this mechanical pathway in the mammalian intestine by ectopic application of forces to intestinal crypts was shown to trigger increased proliferation and concomitant hyperplasia ^29^. Importantly, mechano-sensitive β-catenin/TCF signaling downstream of E-cadherin functions beyond the regulation of cell proliferation, and fulfills an evolutionary conserved role in cellular differentiation that underlies tissue specification during embryonic development ^82– 85^. E-cadherin mechanotransduction was recently also linked to the control of cell metabolism through LKB1-dependent junctional recruitment and activation of AMPK and concomitant ATP production ^28^. Given the importance of metabolic regulation for the support of cell proliferation ^86^, this could provide additional mechanisms by which force transduction by AJs impacts tissue growth.

Tissue growth is determined not only by the rate of proliferation but also by the loss of cells from the tissue that is similarly under control of mechanical signals. Epithelial crowding and consequent mechanical compression were demonstrated to trigger epithelial extrusion, driving loss of both apoptotic and live cells from epithelial tissues ^87– 91^. The stretch-sensitive calcium channel Piezo1 was identified as the main responsible mechano-transducer in this process ^87^. Since modulation of E-cadherin adhesions can trigger epithelial delamination ^92, 93^ and the distribution of tensile forces on E-cadherin junctions has been implicated in apical extrusion of apoptotic cells ^94, 95^, it will be interesting to test whether mechanotransduction by E-cadherin junctions plays a role in density-dependent cell extrusion as well.

## Orienting cell divisions by intercellular forces

Shaping of tissues can be directed by the orientation of cell divisions and subsequent positioning of daughter cells. Division orientation is specified by the position of the mitotic spindle, which is controlled by pulling forces on astral microtubules that link the mitotic spindle to the cell cortex ^96^. Across metazoan species, including *Xenopus*, zebrafish, *Drosophila*, and mammals, it has been observed that epithelial divisions align with anisotropic tensile forces ^97– 100^. This serves to direct tissue elongation ^65, 100, 101^ and to dissipate high levels of anisotropic tissue tension that may arise during morphogenesis ^97, 98, 101^.

**Figure 4.**
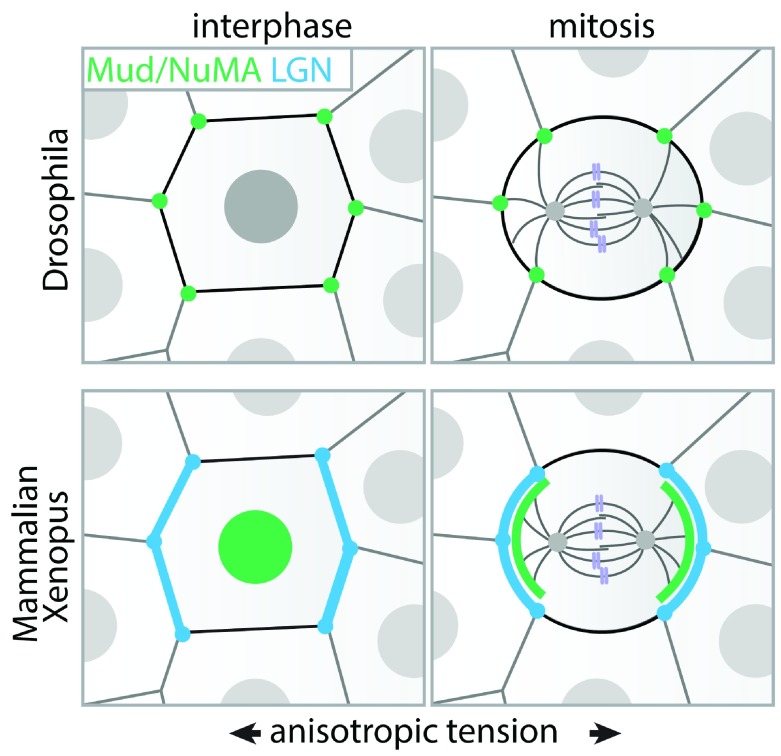
Regulation of cell division orientation by intercellular forces. In the *Drosophila* pupal notum (top panel), the dynein-associated protein Mud is recruited to tricellular junctions (TCJs) during the G _2_ phase of the cell cycle. In this way, in mitosis TCJs form cortical anchor points for astral microtubules that generate dynein-dependent pulling forces to orient the mitotic spindle. The distribution of TCJs aligns with cell shape and mechanical stress orientations within the epithelium, which is preserved upon mitotic rounding. In mammalian and *Xenopus laevis* epithelia (bottom panel), the Mud homologue NuMA is retained within the nucleus during interphase. Instead, the NuMA-interacting protein LGN, which directly interacts with E-cadherin, is recruited to junctions that are under elevated tension, leading to its polarization in mechanically stretched epithelial monolayers. Following mitotic entry and nuclear breakdown, the E-cadherin/LGN complex directs the recruitment of NuMA to cell–cell adhesions and thereby aligns cell divisions with the tension axis, which can overrule the interphase long axis of the cell in orienting division. Although this is not directly apparent in mammalian epithelia ^25, 107^, LGN is enriched at TCJs in *Xenopus* epithelia ^113^, implying that the tension-dependent recruitment of LGN by E-cadherin also provides a mechanism to align division with the position of TCJs.

Initially, tension-oriented cell division was considered to be primarily a consequence of cell elongation along the tension axis ^65, 97, 98, 101^. This idea is supported by the “long axis rule”, postulated by Oscar Hertwig already in the late 19th century, stating that cells tend to divide along their longest axis. However, in epithelial monolayers, cell shape information is typically compromised by mitotic rounding. It was revealed in the *Drosophila* pupal notum how epithelial cells are able to preserve information of their interphase shape during mitosis ^102^. Tricellular junctions (TCJs), specialized adhesions at sites where three (or more) cells meet, recruit microtubule force generators via the dynein-associated protein Mud. The distribution of TCJs aligns with cell shape and mechanical stress orientation within the tissue and this spatial information is retained upon mitotic rounding ( Figure 4) ^102^. Future experiments may answer whether Mud recruitment to TCJs is mechanically controlled, as TCJs are sites of increased intercellular tension ^103^. Similar to *Drosophila*, the Mud homologue NuMA is required for cell divisions to align with the direction of tension in stretched mammalian epithelia ^104^. However, in contrast to Mud, NuMA is retained within the nucleus during interphase ^105– 107^. Instead, during interphase in mammalian epithelia, the NuMA-interacting protein LGN is recruited to E-cadherin junctions in a tension-dependent manner, thereby localizing in a polarized fashion under conditions of anisotropic junctional tension ^25^. Following mitotic entry and nuclear breakdown, the E-cadherin/LGN complex directs the recruitment of NuMA to cell–cell adhesions and enables orientation of the mitotic spindle along the axis of tissue tension ( Figure 4). LGN competes for E-cadherin binding with p120-catenin ^107, 108^, which may underlie its force-dependent recruitment to cell–cell junctions as p120-catenin was recently shown to relocalize from cell–cell junctions to the cytosol upon increased intercellular tension ^109^. Application of a low level of stretch to mammalian epithelial monolayers showed not only that force-dependent recruitment of LGN to E-cadherin is required to align divisions along the tension axis but also that this mechanism can overrule cell shape in determining division orientation ^25^. Shape-uncoupled orientation of division by tissue tension was recently also shown *in vivo* in the *Drosophila* mesectoderm and follicle epithelium, in which cells divide preferentially along the direction of tissue expansion that is perpendicular to the interphase long axis ^110, 111^. In these cells, this occurs independently of either TCJs or LGN/NuMA and instead involves anisotropic remodeling of the actomyosin cytoskeleton and cortical mechanics that may affect the ability of force generators to exert forces on the mitotic spindle ^110, 111^. Because of the role of E-cadherin in force-dependent remodeling of junctional actin, it will be interesting to explore whether regulation of the actomyosin cytoskeleton contributes to the ability of E-cadherin to align cell divisions with anisotropic tensile forces.

## Mechanical forces underlying cell–cell intercalation

In addition to being transmitted by AJs and transduced into an intracellular response, mechanical forces can regulate tissue shape by remodeling of cell–cell contacts through force-dependent regulation of AJ turnover ^109^. This occurs most prominently during cell intercalation, which drives convergence of the tissue along one axis and extension along the orthogonal axis ^112^. Cell rearrangements underlying intercalation are driven by shrinkage and disassembly of junctions perpendicular to the axis of extension, followed by formation of junctions with new neighbors. During *Drosophila* germband extension, shrinkage of dorsoventral junctions is controlled by polarized induction of actomyosin contractility ^114– 116^. The underlying increase in internalization of the cadherin complex in part involves Y667 phosphorylation of β-catenin (Y654 in mammals) ^117, 118^, an event that was recently shown to be directly regulated by tensile force ^82^. A possible explanation of the opposite effects of myosin-generated tension on cadherin junctions, either junction shrinkage or junction reinforcement (discussed earlier), may lie in the force orientation with respect to the junction. During junction shrinkage, forces parallel to the cell–cell contact orientation result in shear stress on the junction complex, which decreases E-cadherin levels at the junction ^119^. In contrast, force that is oriented perpendicular to anterior–posterior junctions was shown to increase junctional E-cadherin levels ^119^. While E-cadherin levels decrease in shrinking junctions, the tension-sensitive recruitment of the actin regulators Vinculin, Ajuba, and AIP1 is increased, enabling the junction to cope with the elevated mechanical load ^119– 121^. After shrinkage of dorsoventral junctions, the germband extends upon resolving the intermediate multicellular vertices and elongation of anterior–posterior junctions. For AJs to withstand the high level of tension at multicellular vertices and for subsequent vertex resolution, the force-sensitive recruitment of Ajuba is essential ^121^. Intriguingly, junction elongation does not happen merely as a result of tissue relaxation but instead is actively controlled by actomyosin activity as well ^122, 123^. Clearly, a dynamic interplay between mechanically controlled AJ turnover and strengthening is essential during intercalation, and future studies will likely provide more insights into the role of E-cadherin mechanotransduction in this.

## Future perspectives

AJs have emerged as integral components in the regulation of morphogenesis by mechanical forces. In recent years, molecular details of cadherin mechanotransduction in morphogenetic processes of collective migration, cell division, and cell intercalation have started to be resolved. Future research will likely shed more light on how cadherin mechanotransduction impacts other processes that regulate tissue development and homeostasis, such as the control of cell shape, differentiation, and delamination ^2, 22, 92, 124^. Several important questions remain just at the start of investigation, for instance how cells discriminate between forces of different magnitudes and timescales. Different timescales add the conundrum that the short-term responses of cells to intercellular forces (for example, cell shape change or Vinculin recruitment to adhesion sites) function to dissipate force and therefore potentially prevent long-term responses ^125^. Moreover, it remains unanswered whether different force-dependent constituents of the AJ can be present simultaneously within the same cadherin complex, and mutual exclusivity is likely to exist (for example, between proteins competing for α-catenin binding). Understanding how the different components of cadherin mechanotransduction may be responsive to distinct magnitudes and types of force ^126^ will further help to explain why cells may adopt different, sometimes opposing, mechano-responses through cadherin junctions (for example, junction reinforcement or junction remodeling).

Although we have focused primarily on AJs, cell–cell contacts contain additional adhesion complexes (that is, tight junctions and desmosomes) that are exposed to external mechanical force ^127^, adopt tension-sensitive changes in their composition ^128^, and contribute to the transduction of intercellular forces ^39^. Moreover, intercellular forces can be sensed by mechano-sensing complexes that respond to membrane deformations, a mechanism used by mechano-sensitive ion channels that adopt open and closed conformations depending on local force-induced membrane deformations ^129, 130^. So far, research on mechanotransduction has predominantly approached the various mechano-sensitive complexes as isolated units. To properly understand cellular responses to forces, it will be pivotal to know how they cooperate. Interplay further exists between AJs and mechano-sensitive complexes that are not localized at cell–cell contacts, such as focal adhesions that contain numerous mechano-sensitive components that are in part shared with the AJ ^6^. Mechanotransduction by both adhesion complexes can converge on similar transcription factors and may cooperate in the activation of signaling pathways as has been shown for the activation of β-catenin ^77^. In coming years, unraveling the interplay between these different mechano-sensitive complexes will be instrumental in understanding the variety of cellular behaviors that are controlled by mechanical force.

Finally, it is becoming increasingly apparent that mechanical signals that cells receive from their environment continuously crosstalk with biochemical signals, such as growth factors and hormones. Not only can both impinge on the same intracellular signaling cascades, but several growth factor receptors are also directly (for example, Notch ^131, 132^) or indirectly (for example, EGFR ^21^ and insulin receptor ^133^) regulated by mechanical force. Moreover, biochemical signaling pathways may feedback to modulate the cellular mechano-response ^134^. A better comprehension of the dynamic interplay between mechanical and biochemical signaling will be imperative to understand the complexities of animal development.
